# Supplementary material for: Myosin-dependent short actin filaments contribute to peripheral widening in developing stereocilia
Source: Nat Commun. 2025 Jul 1;16:5835. doi: 10.1038/s41467-025-60976-y (PMC12215623; doi:10.1038/s41467-025-60976-y)
Supplement: Supplementary file 1 — Supplementary information [file 41467_2025_60976_MOESM1_ESM.pdf]

Myosin-dependent short actin filaments contribute to peripheral widening in developing stereocilia

Xiayi Liao[1], Chun-Yu Tung[1], Jocelyn F. Krey[2,3], Ghazaleh Behnammanesh[4], Joseph A. Cirilo Jr[5], Mert Colpan[6], Christopher M. Yengo[5], Peter G. Barr-Gillespie[2,3], Jonathan E. Bird[4], Benjamin J. Perrin[1*]

[1] Department of Biology, Indiana University, Indianapolis, IN, USA.
[2] Oregon Hearing Research Center, Oregon Health & Science University, Portland, OR, USA.
[3] Vollum Institute, Oregon Health & Science University, Portland, OR, USA.
[4] Department of Pharmacology and Therapeutics, University of Florida, Gainesville, FL, USA.
[5] Department of Cellular and Molecular Physiology, Penn State College of Medicine, Hershey, PA, USA.
[6] Department of Cellular and Molecular Medicine, The University of Arizona, Tucson, AZ, USA.

*Corresponding Author: Dr. Benjamin Perrin. Email: bperrin@iu.edu.

Supplementary Information includes supplementary figures 1-7.

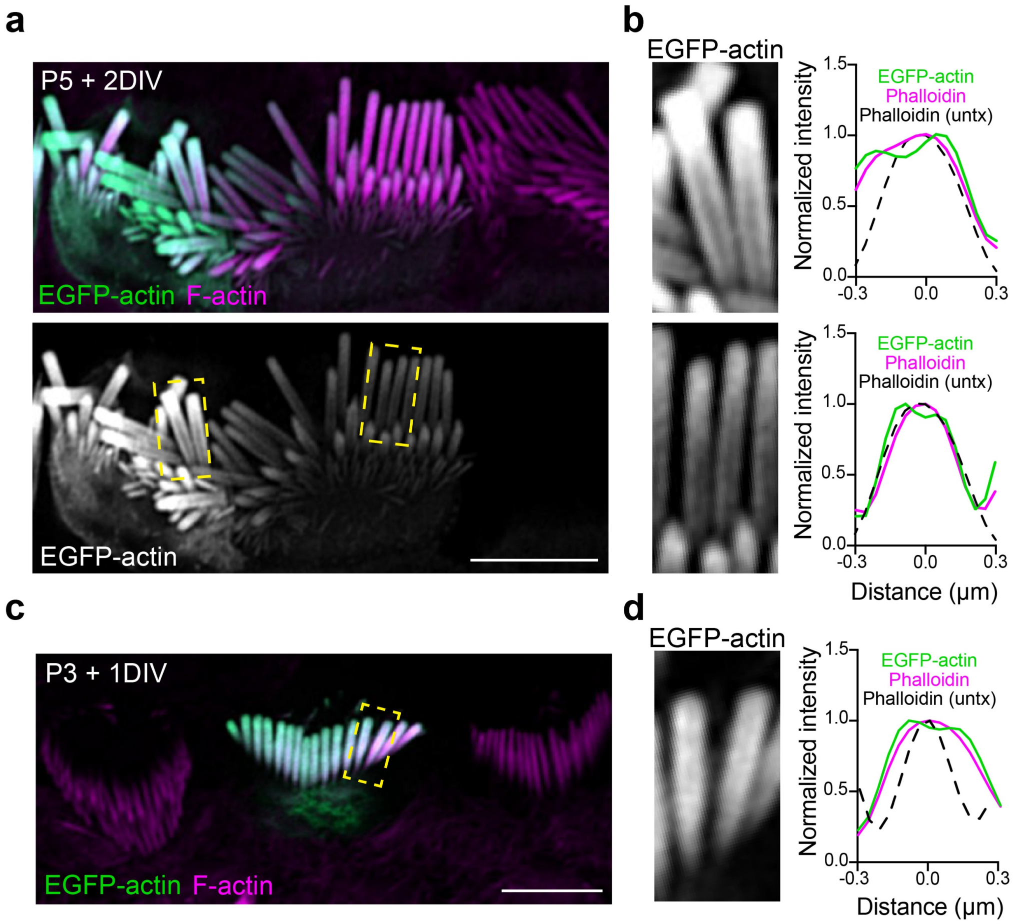

Supplementary Fig. 1: EGFP-incorporation pattern in IHCs with prolonged transfection or at early postnatal age.

a, P5 IHCs 48 hours (2 DIV) after transfection with EGFP-actin (green and grey). F-actin is stained by phalloidin (magenta) to show stereocilia. **b**, Magnified insets from (**a**, yellow dashed boxes) showing stereocilia with higher or lower level of EGFP-actin (grey). Line scans drawn perpendicular to stereocilia shafts include the distribution of EGFP-actin (green) and F-actin (magenta) in transfected cells compared to F-actin (black dashed lines) in adjacent untransfected cells (untx). **c**, P3 IHCs 18 hours (1 DIV) after transfection with EGFP-actin (green). F-actin is stained by phalloidin (magenta). **d**, Magnified inset from (**c**, yellow dashed box) showing EGFP-actin (grey) and line scans drawn perpendicular to stereocilia shafts. Line graph includes the distribution of EGFP-actin (green) and F-actin (magenta) in transfected cells compared to F-actin (black dashed lines) in adjacent untransfected cells (untx). The experiments were repeated three times with similar results. Scale bars represent 5 µm. DIV: days in vitro.

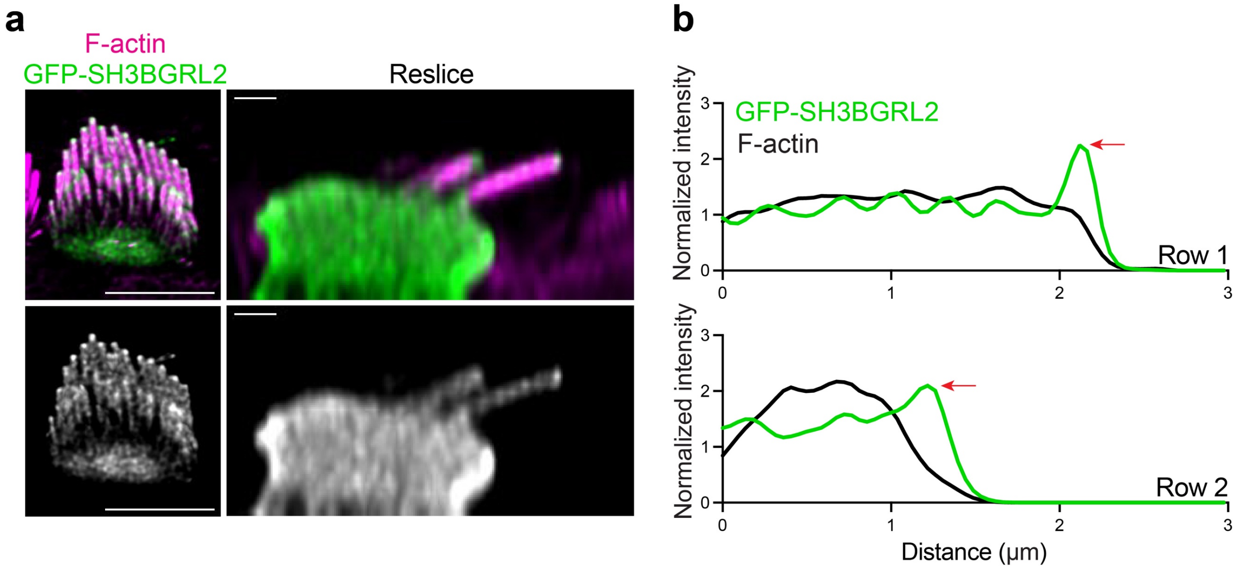

Supplementary Fig. 2: Localization of transfected EGFP-SH3BGRL2 in IHCs.

a, Representative images of EGFP-SH3BGRL2 distribution in P5 IHCs 18 hours post transfection. Left panels are 2D projections of x-y slices (scale bar represents 5 μm). Right panels are projections of x-z reslices to show the side view of stereocilia (scale bar represents 1 μm). The experiments were repeated three times with similar results. **b,** Fluorescence distribution of EGFP-SH3BGRL2 and phalloidin stained F-actin measured on the line scan of a stereocilium in (**a**). Red arrows indicate that the intensity of EGFP-SH3BGRL2 reaches near its peak at stereocilia tips.

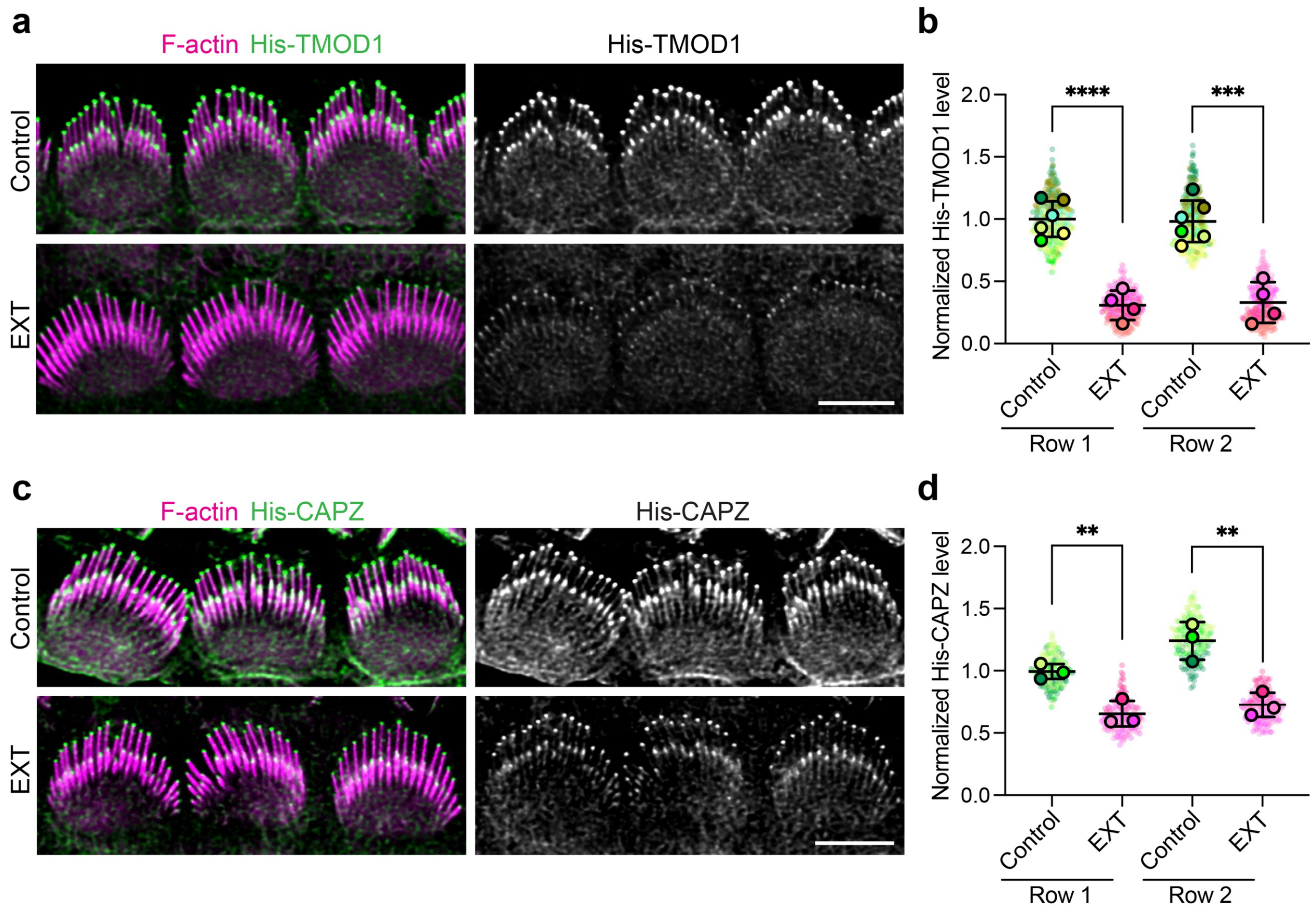

Supplementary Fig. 3: Tip filaments are separate from the stereocilia core filaments.

a, His-TMOD1 staining (green, grey) in P5 IHCs after high salt extraction (EXT); F-actin was stained with phalloidin (magenta). **b**, Quantification of His-TMOD1 level from row 1 and row 2 stereocilia tips. The fluorescence intensity was normalized to the average fluorescence intensity of row 1 control treatment. **c,** His-CAPZ staining (green, grey) in P5 IHCs after high salt extraction; F-actin was stained with phalloidin (magenta). **d,** Quantification of His-CAPZ level from row 1 and row 2 stereocilia tips. Smaller circles represent stereocilia; larger open circles represent cochleae (N). *P* values for two-tailed unpaired *t* tests comparing N are indicated (**, P =0.0079 for row 1 comparison and P =0.0078 for row 2 comparison; ***, P = 0.0003; ****, P < 0.0001). Results from 6 cochleae for His-TMOD1 labeling and 3 cochleae for His-CAPZ labeling were respectively averaged and plotted ± SD. Scale bars represent 5 μm.

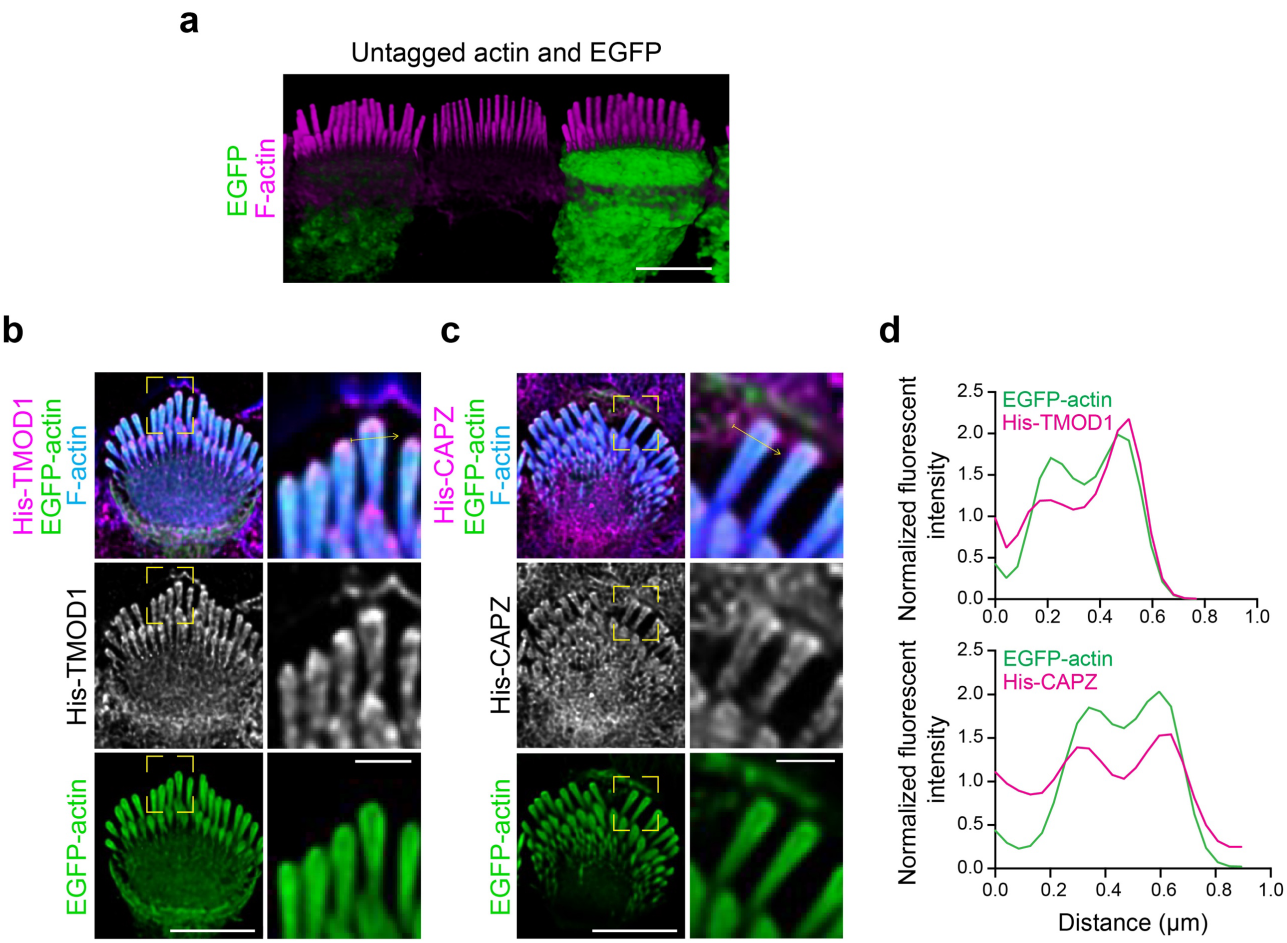

Supplementary Fig. 4: Untagged actin overexpression promotes stereocilia widening.

a, Representative image showing a 3D reconstruction of P5 IHCs transfected by Actin-IRES-EGFP. Transfected IHCs, identified by cytoplasmic EGFP, exhibit wider stereocilia compared to a neighboring untransfected IHC. F-actin is stained by phalloidin (magenta). **b-c,** Representative images showing His-TMOD1 (**b**) or His-CAPZ staining (**c**) (magenta, grey) in widened stereocilia (blue) from IHCs transfected with EGFP-actin (green) (scale bar represents 5 μm). Regions marked by yellow boxes are magnified to the right panels (scale bar represents 1 μm). The yellow arrows indicate line scans graphed in (**d**). **d,** The fluorescence distribution of His-TMOD1 or His-CAPZ (magenta) with EGFP-actin (green). The fluorescence intensity was normalized to the average fluorescence intensity of each label. The experiments were repeated three times with similar results. In all cases cells were analyzed 18 hours post transfection.

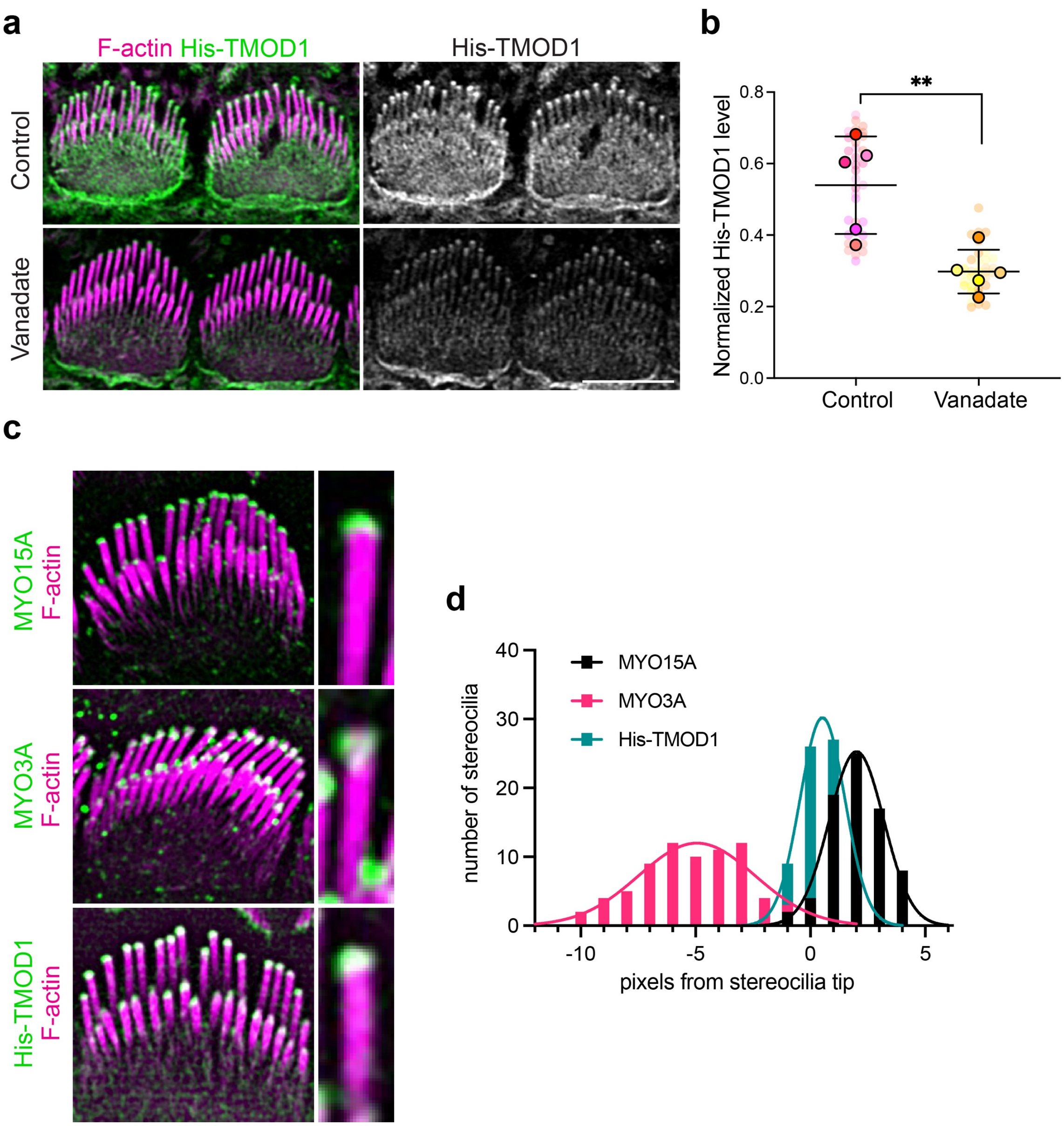

Supplementary Fig. 5: Tip filaments are likely stabilized or produced by myosins.
a, His-TMOD1 staining (green, grey) in P5 IHCs incubated with or without vanadate. **b,** Quantification of His-TMOD1 level at stereocilia tips in vanadate-treated and untreated IHCs. Smaller circles represent stereocilia; larger open circles represent cochleae (N). *P* values for two-tailed unpaired *t* tests are indicated based on N (**, $P = 0.0069$). Results from 5 cochleae were averaged and plotted ± SD. **c,** Representative lattice SIM images showing the localization of endogenous MYO15A, MYO3A, and His-TMOD1 (green) in IHC bundles. The magnified insets show the localization of each protein at row 1 stereocilia tips compared to phalloidin-stained F-actin (magenta). **d,** A frequency histogram showing the pixel offset of MYO15A (black), MYO3A (red) and His-TMOD1 (blue) from the actin core. The histogram of each probe is fitted in a Gaussian curve. Mean offsets for peak of the Gaussian curves: MYO15A, 62 nm; MYO3A, -153 nm; His-TMOD1, 16 nm. R-squared value of the fit: MYO15A, 0.981; MYO3A, 0.904; His-TMOD1, 0.996. The stereocilia tip was defined as being the point where phalloidin intensity reached the average value in the tip region. Results were collected from 75 stereocilia for each probe. Scale bars represent 5 μm.

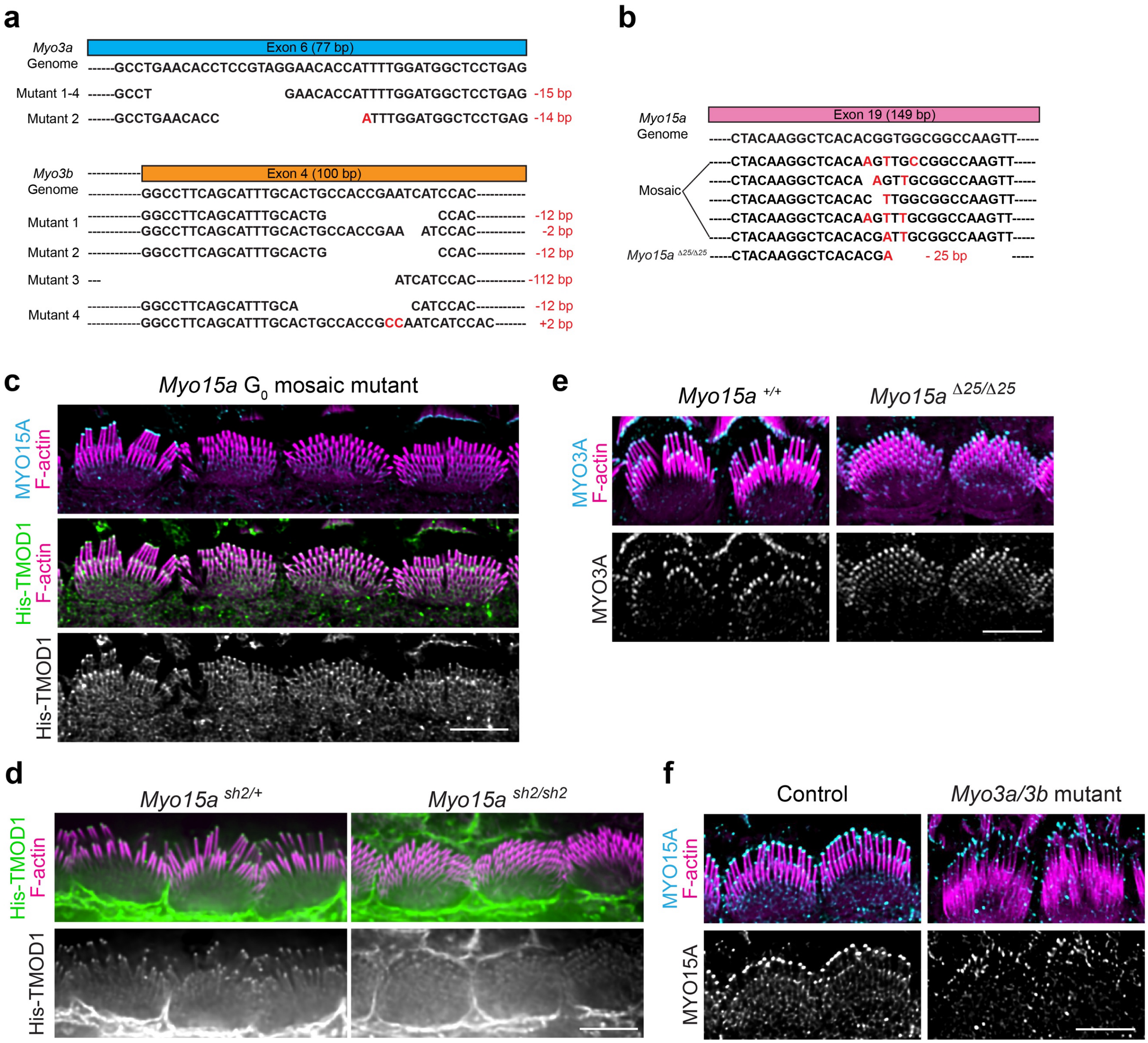

Supplementary Fig. 6: Characterization of mutant *Myo3* and *Myo15a* alleles.

a-b, Schematics showing mutant alleles detected by nanopore sequencing of genomic DNA from pups that were mutated by CRISPR/Cas9 gRNAs targeting *Myo3a* and *Myo3b* concurrently (**a**) or *Myo15a* (**b**) that were delivered to mouse embryos by the i-GONAD method. **c,** Mutant *Myo15a* exhibiting a mosaic phenotype. MYO15A (cyan) and His-TMOD (green, grey) at highest at the tips of the hair cell at left that retains normal stereocilia lengths, but His-TMOD1 is reduced at the tips of stereocilia on neighboring hair cells that are short and lack MYO15A. **d,** His-TMOD1 staining (green, grey) in P4 IHCs from mice heterozygous or homozygous for the *sh2* loss-of-function mutation. Homozygous mutants have short stereocilia with reduced His-TMOD1 staining. **e,** MYO3A immunostaining (cyan, grey) of either wildtype or *Myo15a*$^{\Delta 25/\Delta 25}$ P4 IHC stereocilia. **f**, MYO15A immunostaining (cyan, grey) of either wildtype or *Myo3a;Myo3b* mutant P4 IHC stereocilia. F-actin was stained with phalloidin (magenta) in (**c-f**).

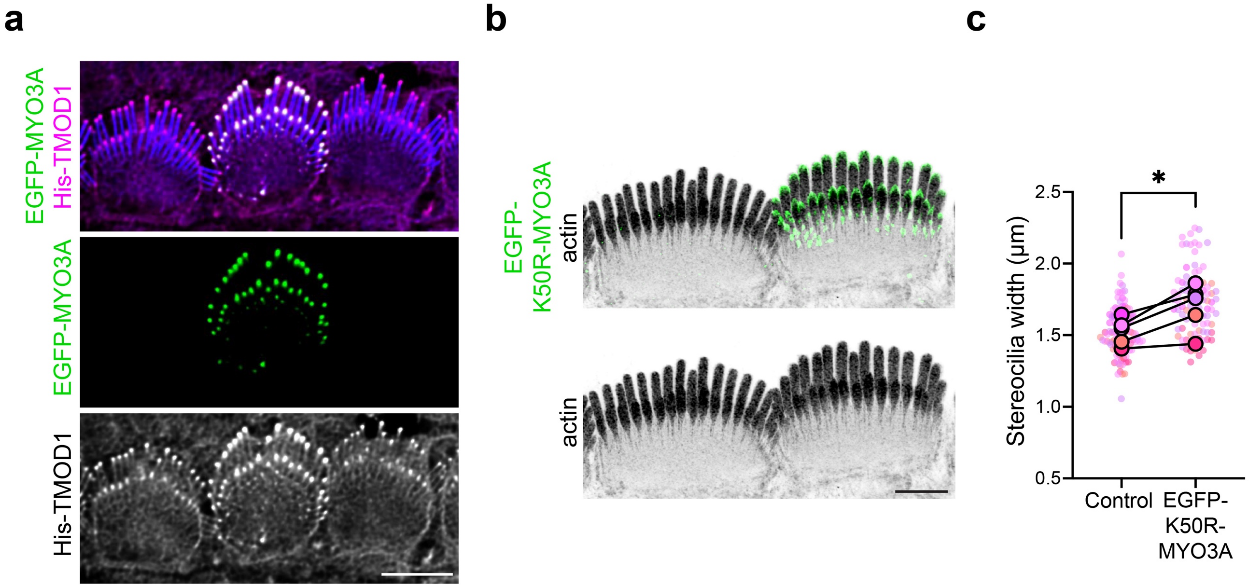

Supplementary Fig. 7: Overexpression of EGFP-MYO3A and stereocilia widening.
a, Representative images comparing His-TMOD1 staining (magenta, grey) in P5 IHCs 18 hours after transfection with wild-type (WT) EGFP-MYO3A (green) compared to neighboring untransfected cells. F-actin is stained with phalloidin (blue). **b,** Representative expansion microscopy images of an EGFP-K50R-MYO3A transfected IHC adjacent to an untransfected IHC at P5. EGFP-K50R-MYO3A (green) was stained with an antibody to EGFP, and actin (grey) was stained with anti-γ-actin antibody. **c**, Quantification of stereocilia width in EGFP-K50R-MYO3A transfected cells and untransfected cells. Smaller circles represent individual stereocilia and larger open circles represent cochleae. Results were collected from 5 cochleae. *P* values for two-tailed paired *t* tests are indicated (*, $P = 0.0157$), comparing averages of cochleae. Scale bars represent 5 µm in (**a**) and 10 µm in (**b**).
